# Codesigning person‐centred quality indicators with diverse communities: A qualitative patient engagement study

**DOI:** 10.1111/hex.13388

**Published:** 2021-12-02

**Authors:** Kimberly Manalili, Fartoon M. Siad, Marichu Antonio, Bonnie Lashewicz, Maria J. Santana

**Affiliations:** ^1^ Department of Community Health Sciences University of Calgary Calgary Alberta Canada; ^2^ ActionDignity Calgary Alberta Canada; ^3^ Department of Pediatrics University of Calgary Calgary Alberta Canada

**Keywords:** codesign, diverse communities, participatory action research, patient engagement, person‐centred care, qualitative research, quality indicators

## Abstract

**Introduction:**

Effective engagement of underrepresented communities in health research and policy remains a challenge due to barriers that hinder participation. Our study had two objectives: (1) identify themes of person‐centred care (PCC) from perspectives of diverse patients/caregivers that would inform the development of person‐centred quality indicators (PC‐QIs) for evaluating the quality of PCC and initiatives to improve PCC and (2) explore innovative participatory approaches to engage ethnocultural communities in qualitative research.

**Methods:**

Drawing on participatory action research methods, we partnered with a community‐based organization to train six ‘Community Brokers’ from the Chinese, Filipino, South Asian, Latino‐Hispanic, East African and Syrian communities, who were engaged throughout the study. We also partnered with the provincial health organization to engage their Patient and Family Advisory, who represented further aspects of diversity. We conducted focus group discussions with patients/caregivers to obtain their perspectives on their values, preferences and needs regarding PCC. We identified themes through our study and engaged provincial stakeholders to prioritize these themes for informing the development of PC‐QIs and codesign initiatives for improving PCC.

**Results:**

Eight focus groups were conducted with 66 diverse participants. Ethnocultural communities highlighted themes related to access and cost of care, language barriers and culture, while the Patient and Family Advisory emphasized patient and caregiver engagement. Together with provincial stakeholders, initiatives were identified to improve PCC, such as codesigning innovative models of training and evaluation of healthcare providers.

**Conclusion:**

Incorporating patient and community voices requires addressing issues related to equity and understanding barriers to effective and meaningful engagement.

**Patient or Public Contribution:**

Patient and public engagement was central to our research study. This included partnership with a community‐based organization, with a broad network of ethnocultural communities, as well as the provincial health service delivery organization, who both facilitated the ongoing engagement of diverse patients/caregiver communities throughout our study including designing the study, recruiting participants, collecting and organizing data, interpreting findings and mobilizing knowledge. Drawing from participatory action research methods, patients and the public were involved in the codesign of the PC‐QIs and initiatives to improve PCC in the province based on the findings from our study.

## 1 INTRODUCTION

Person‐centred care (PCC) is a model of holistic care that engages patients and caregivers in healthcare and recognizes the importance of providing personalized care that affirms the dignity of the patient.^1^ Furthermore, PCC is an approach to care that promotes the patient and caregiver perspective, acknowledging that patients are experts in their own health and experience with their illness.^1^ While evidence suggests that PCC is key to improving the quality of healthcare delivery, PCC is not routinely measured or evaluated from the patient and caregiver perspective.^2^ In particular, members of communities that tend to be marginalized do not tend to be included in assessments related to quality of care.^3^ As such, improvements in healthcare quality may not take into account what matters most to those who may experience barriers to care. Understanding the important aspects of PCC from the perspective of patients and caregivers can inform the development of person‐centred quality indicators (PC‐QIs), which help to guide improvements in the care provided to patients. The development of PC‐QIs informed by patients and caregivers can provide healthcare providers, policymakers and organizations with the information they need to identify measurable gaps in the delivery of PCC and target needed action to improve the quality and delivery of care for all.^1^, ^4^

A patient‐engaged research approach was central to our goal of developing the themes for the PC‐QIs. This approach is about meaningfully engaging patients and caregivers towards ensuring that PC‐QIs truly reflect what matters most to them. Despite research efforts to engage patients by using participatory methods,^5^, ^6^ engaging diverse patients, family caregivers and communities remains challenging,^7^, ^8^ considering that diversity includes involving those from different ethnic and racial communities, language groups, various ages, abilities, geographic location (e.g., rural), sexual orientations and gender identities. In particular, linguistic, cultural and economic barriers hinder participation in patient engagement research and healthcare policy.^9^ Thus, the need to include diverse perspectives and distinct healthcare system experiences, particularly among immigrant and newcomer communities, remains pressing.^10^ Diverse perspectives and unique experiences include different care preferences and expectations, as well as varied understandings of the healthcare system and persistent disparities in care and health outcomes. Such factors can have negative implications not only for access to care but also for patient safety, experiences and outcomes—all of which challenge the efforts of healthcare systems towards delivering PCC.^11^, ^12^ Innovative and equitable approaches for engagement are needed towards ensuring that the voices of diverse—and often marginalized—communities are incorporated as part of how healthcare systems assess PCC.

Our aim to meaningfully engage diverse patients and communities in the development of the PC‐QIs as well as related initiatives to improve PCC was advanced through two objectives: (1) to identify key themes of PCC, from the perspectives of diverse patients and caregivers, which will inform the development of PC‐QIs as well as initiatives to improve PCC, and (2) to use innovative participatory approaches to engage ethnocultural and immigrant communities in qualitative patient‐engaged research. This study is part of a larger programme of research at the University of Calgary to develop and implement PC‐QIs for system‐level application in Canada^2^, ^10^, ^13^, ^14^, ^15^ and has been approved by the University Health Research Ethics Boards (REB15‐2846) at the University of Calgary.

## 2 METHODS

### 2.1 Design

To address our research questions and study objectives, an exploratory–descriptive qualitative study design^16^ was chosen with the goals of exploring the patient and caregiver lived experience with healthcare and identifying similarities and differences across groups regarding healthcare preferences, needs and values. Our focus on attaining the perspectives of diverse communities seeks to address the paucity of literature in this area, consistent within exploratory approach.^17^ The descriptive element of our approach promotes an application of our findings towards contributing to quality improvement and change in healthcare settings.^18^, ^19^ The theoretical anchoring for our study design was a transformative paradigm, which is rooted in the principle that knowledge is not neutral, but rather reflects the social and power relationships that exist in society.^20^ A transformative paradigm guided us to centre the experiences of traditionally marginalized communities, analyse the power differentials that may lead to marginalization and use research methods drawn from participatory action research practices.^21^

### 2.2 Research partnerships

Guided by a transformative paradigm, the study was designed with community research partners who would collaborate on achieving our study aim to meaningfully engage diverse patients and communities in the development of the PC‐QIs as well as related initiatives to improve PCC. We purposely partnered with specific organizations that are based in Alberta, who share our vision for attaining our study aims and objectives, and who we felt would be the most appropriate to partner with (i.e., interest in making system‐level healthcare improvements and experience). Our study team at the University of Calgary partnered with ActionDignity Society, a community‐based organization in Calgary, Alberta, Canada, who employ a ‘Cultural/Community Broker’ approach to engage ethnocultural communities in issues related to systems and policy change.^22^ ActionDignity provides specialized training to Community Brokers (also known simply as ‘Brokers’) to effectively engage communities and act as a liaison with service providers/institutions. In addition, we partnered with Alberta Health Services (AHS), the provincial health service delivery organization, to collaborate with their Patient and Family Advisory Group, who would represent further aspects of diversity in Alberta.

### 2.3 Community Brokers

Consistent with participatory action principles, we engaged six Community Brokers who were trusted, well‐connected community members, and who understood the assets, as well as the barriers and challenges, faced by ethnocultural communities. The Brokers included two men and four women who had an interest in learning about healthcare research. The Brokers represented communities within the province of Alberta that are extensive and long‐standing (Chinese, South Asian and Filipino) as well as growing and emerging (Latino‐Hispanic, East African and Syrian).^23^

In collaboration with our partners, data were collected through focus group discussions (FGDs) to invite a conversational exchange about diverse experiences and perspectives. The Brokers received training by the University of Calgary research team and ActionDignity staff about how to recruit participants, conduct and transcribe the FGDs, support analysis, interpretation and share findings through a series of four evening workshops at the ActionDignity office. Our research team provided ongoing mentorship over the course of 9 months. Two of the six Brokers had previous experience conducting research with ActionDignity, and all six had extensive experience working in community settings. The Brokers were employed in various settings, including social/community work (four), nursing student (one) and as a lab technician (one). The Brokers completed a confidentiality agreement regarding the collection and management of participant data. Following the completion of the study, each Broker received a training certificate and an honorarium of $1500.

**Figure 1:**
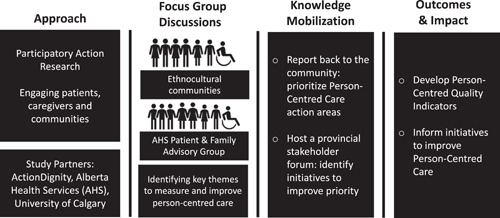
Overview of our study. Our study approach included participatory action research and engagement of patients, caregivers and communities throughout the study. Establishing our research partnership was the first step in our research process. Together as research partners, we conducted focus group discussions with ethnocultural communities (through ActionDignity networks) and other diverse communities in Alberta (via the AHS Patient and Family Advisory Group). The findings from the focus group discussions were shared with provincial stakeholders and mobilized through two events: (1) Reporting back to the community to prioritize the person‐centred care (PCC) themes identified by participants as ‘action areas’ to improve PCC and (2) a provincial stakeholder forum, where specific initiatives were identified to improve the prioritized areas of PCC. The outcomes and impact of our research include the development of person‐centred quality indicators to evaluate PCC, as well as initiatives to be implemented for improving the delivery of PCC

Along with our partners, we also invited provincial health stakeholders (patients, caregivers, community members, researchers, policymakers, health service delivery organizations) to respond to the findings from the FGDs to codesign initiatives to improve PCC. Figure 1 presents an overview of our study partners, activities, outcomes and impact.

### 2.4 Focus groups with ethnocultural communities: Participant recruitment and inclusion criteria

Community Brokers led ethnocultural community participant recruitment using a mixed sampling approach to recruit 6–10 members from their respective ethnocultural communities of origin while striving for maximum variation with regard to participant self‐identified gender, age and health services accessed. Mixed sampling involved recruiting through the ways in which community members commonly engaged in their community. This entailed snowball sampling of individuals who would be interested (e.g., former healthcare professionals in their home country), convenience sampling through community organizations and events and sampling using social media groups and ethnic media (radio) in the native language of the community and/or in English. In some cases, the Broker was familiar with participants, having met them previously.

To be included, participants had to be from the target communities; willing to share their experiences; fluent in the language in which the FGDs were conducted; adults ≥18 years of age; be patients or family members of patients who have ever accessed health services in Alberta (e.g., family doctor visits, emergency department visits, laboratory services, long‐term care, etc.); not employed by AHS; and have lived in Canada for 2–10 years. The requirement for being a relatively new immigrant was set, given our study focus on including ethnoculturally diverse groups of participants.

### 2.5 Focus groups with (health organization name) Patient and Family Advisory members: Participant recruitment and inclusion criteria

AHS used convenience sampling to recruit participants from among their Patient and Family Advisory Group, comprised of about 30 members who are highly engaged and familiar with the Albertan healthcare system. Members of this group represented long‐term residents of Canada (>10 years of residency), different age groups, racialized communities, urban and rural communities, Indigenous communities and those from the 2SLGBTQIA (2‐Spirit, Lesbian, Gay, Bisexual, Transsexual, Queer, Intersex, Asexual) community.

The University of Calgary and ActionDignity research members worked together to coordinate recruitment. Letters of introduction and consent forms, written in English, were provided to all potential participants and translated as needed by the Brokers. All potential participants completed forms to provide sociodemographic information (i.e., gender, age, occupation, education, (dis)ability, health services accessed, years lived in Canada, country of origin).

### 2.6 Data collection

FGDs with the ethnocultural communities were conducted in person in the community (e.g., Colombian Consulate/nonprofit meeting spaces, residential settings) or at the ActionDignity office according to participants' comfort and preferences. The FGDs conducted with members of the AHS Patient and Family Advisory Group took place at an AHS office during a regularly scheduled advisory group meeting. A semistructured interview guide was used (Supporting Information Appendix A). Our interview guide was designed to prompt participants to share their experiences with the Alberta healthcare system. Participants were asked about specific factors that may have shaped their experiences and what they value in their care. The interview guide was previously pilot‐tested with a patient researcher and the Brokers and refined based on their feedback to expand on potential probing questions.

The Brokers facilitated the focus groups with ethnocultural communities, with notetaking support from research volunteers who they recruited. The volunteers were fluent in the language in which each FGD was conducted. The FGDs for the Chinese, Filipino, Syrian and Latino‐Hispanic communities were conducted in the participants' native language (i.e., Mandarin, Tagalog, Arabic, Spanish, respectively), while the East African and South Asian FGDs were conducted in English due to a diversity of languages spoken among the participants. One or two research team members from either the University of Calgary or ActionDignity attended all FGDs to provide support to the Brokers and serve as note‐takers in cases where no research volunteer was available. FGDs with the AHS Patient and Family Advisory Group were conducted by one researcher from the University of Calgary (woman, PhD) and another from ActionDignity (man, MD), each of whom conducted one FGD. Both researchers had extensive experience conducting FGDs and had no previous relationships with participants. Members of the research team came from the Filipino, Somali and Spanish communities, and were able to provide language support/and or help to interpret data, based on an understanding of/experience with these cultural groups. Following each FGD, the research team met to debrief and document how the discussion went and identify preliminary themes.

### 2.7 Ethical considerations

Ethical considerations for research with marginalized populations were made.^24^, ^25^ The Brokers' role in recruiting and facilitating the ethnocultural FGDs would help to ensure cultural sensitivity and comfort of the participants. Before starting each FGD, the informed consent form was reviewed by the facilitators (with a thorough description of the research process) and signed by each participant. Participants were also aware that they may withdraw their participation at any time. Considerations were made to minimize the time burden on participants. Focus groups ran approximately 2 h, of which 75–90 min were dedicated to discussion, with additional time for introductions and socializing. All participants received a $10 gift card to a coffee shop, reimbursement for parking or transit and were provided with refreshments during the discussion. Participants were able to bring their children to the FGDs or to request childcare provided through ActionDignity. All participant data were anonymized and kept confidential within the research team (all research team members, including the Brokers signed confidentiality agreements). Finally, the participatory action nature of this study provides an opportunity to address issues related to social justice that affect marginalized populations.^24^, ^25^

### 2.8 Transcription and translation of the data

All FGDs were audio‐recorded and later transcribed in English. For FGDs conducted in languages other than English, the Brokers simultaneously translated and transcribed the recorded data, following the steps outlined by Hennink.^26^ The participants were offered the opportunity to review the transcripts for comment and correction. Two participants from the AHS Patient and Family Advisory Group undertook this opportunity, but did not suggest any corrections to the transcripts.

### 2.9 Analysis

Sociodemographic data were analysed descriptively using Microsoft Excel to calculate proportions of each variable. Qualitative analysis was conducted by two independent researchers at the University of Calgary, who began by reading the transcripts and field notes to become familiarized with the data.^27^ A semi‐inductive qualitative content analysis approach was used^28^, ^29^ following the principles of constant comparison to uncover underlying patterns among and within groups.^30^ Before this analysis, an initial coding structure was developed based on the preliminary themes identified during the debrief sessions with the research team following each FGD. Nvivo 12 software was used to code and manage the data into additional codes and subcodes as well as make memo notes.^27^ To enhance the trustworthiness,^31^ the analysts met after coding one transcript to compare the codes identified and to discuss any discrepancies. In this meeting, a shared logbook was developed to describe the codes and document any decisions regarding inclusion and exclusion criteria for codes. The analysts met regularly to continue reviewing and refining codes. These codes were then shared with members of the research team for discussion, grouping into larger categories and distilling into broader themes and subthemes until data saturation was reached, where no new themes were observed in the data.^27^

### 2.10 Knowledge mobilization

#### 2.10.1 Report back to the community

Following separate meetings with the Community Brokers and AHS to members check the findings, the themes were further refined and developed as ‘action areas’ for improving PCC. These ‘action areas’ were translated into several languages and shared with study participants, members of ActionDignity and community stakeholders focused on supporting health services for ethnocultural communities (e.g., HIV Community Link, Alberta Health Services Multicultural Health, Punjabi Community Health Services) in February 2017, as a ‘report back to community’ event.

In this meeting, participants discussed and confirmed the findings as well as prioritized the ‘action areas’ deemed most important to measure and to improve. Prioritization was achieved through ‘dotmocracy’, which is a common tool used in community settings to achieve a form of consensus.^32^, ^33^ Each participant was provided with five coloured dots that were used to cast a ‘vote’ for their top five priorities among the 10 action areas presented. The votes were counted to determine the top priorities and discussed with the participants.

#### 2.10.2 Provincial PCC Forum

The findings from the FGDs along with the prioritized action areas were shared at a provincial PCC Forum in March 2017, organized by the O'Brien Institute for Public Health, University of Calgary. Leadership of key provincial stakeholders in healthcare, such as the Ministry of Health, AHS, researchers, patients, community members and organizations, participated in the forum. As part of the forum, a smaller group of participants took part in a workshop, where they were assigned to one group to discuss one of the priority areas for improving PCC. These participants were recruited to encompass a variety of perspectives, including patients/caregivers/community members, researchers, health service providers and policymakers. For their priority areas, each group:

1. Identified opportunities/initiatives for improvements.
2. Discussed considerations for implementing these initiatives.
3. Assessed the feasibility of making improvements, given existing policies and resources.

These discussions were synthesized and documented by the research team. The Community Brokers played a key role in presenting the FGD findings at both the community report‐back event and the provincial forum.

## 3 RESULTS

Eight FGDs, each comprised of 6–10 participants, were conducted with a total of 66 participants from a range of ethnicities/cultures, genders, ages (18–80 years), rural/urban communities and including participants who identified as living with disability(ies), Indigenous and/or identified as 2SLGBTQIA. One FGD was conducted with each of the six targeted ethnocultural communities. Two FGDs were conducted with members of the AHS Patient and Family Advisory Group, who comprised about 30% of the study participants. Slightly more than half of the participants identified as women (55%) and nearly half reported being Canadian Citizens (48%). Participants from the Latino‐Hispanic and East African FGDs were diverse with regard to country of origin. However, participants from India were strongly represented among those in the South Asian group, relative to other countries. Table 1 summarizes the participants' sociodemographic characteristics. No participants attended more than one FGD and no participants refused to participate or discontinued participation in an FGD.

**Table 1:** Demographic characteristics of focus group participants (*N* = 66)

Characteristic	No. (%) of participants
Self‐identified gender, *n* = *62*	
Woman	34 (55)
Man	28 (45)
Age: median (IQR), years, *n* = *60*	40 (30–57)
Living with disability(ies), *n* = *63*	3 (5)
Immigration status, *n* = *63*	
Canadian citizen	30 (48)
Landed immigrant/permanent resident	28 (44)
Student visa	3 (5)
Temporary foreign worker	1 (2)
Open work permit	1 (2)
Length of residence ethnocultural participants: median (IQR), years, *n* = *46*	4.75 (3–8)
Highest level of education, *n* = *62*	
Less than high school	2 (3)
High school completion	8 (13)
Postsecondary certificate/trades	8 (13)
Undergraduate degree	24 (39)
Graduate degree	20 (32)
Employed, *n* = *62*	39 (63)
Student status, *n* = *61*	17 (28)
Community/group, *n* = 66	
Alberta Health Services Patient and Family Advisory	20 (30)
Chinese (Mandarin‐speaking)	8 (12)
East African	8 (12)
Filipino	7 (11)
Latino‐Hispanic	6 (9)
South Asian	9 (14)
Syrian	8 (12)
Health services accessed,^a^ *n* = *63*	
General practitioner/family doctor	57 (90)
Lab services	55 (87)
Walk‐in clinic	47 (75)
Pharmacy/dispensing services	40 (63)
Emergency department	36 (57)
Diagnostic imaging	27 (43)
In‐patient (admitted/overnight stay)	26 (41)
Physiotherapy	21 (33)
Cancer care	7 (11)
Long‐term care	5 (8)
Other (specialist, LGBTQ/youth services, fertility services, dental care)	5 (8)
Palliative care	4 (6)

*Note*: Some responses were missing and not included in this summary.

^a^ Most participants indicated more than one health service accessed.

### 3.1 Key themes to inform PC‐QI development and PCC initiatives

Participants shared their positive and negative perceptions and experiences with healthcare that provided insight into their preferences, needs and values related to healthcare—about what could be considered PCC and what would not. These findings informed the development of PC‐QIs to measure and evaluate PCC as well as the delineation of potential initiatives to improve quality of care. We organized these findings into 10 themes, which are shown and illustrated in Table 2.

**Table 2:** Summary of themes and illustrative quotes

Themes and subthemes	Description of themes and illustrative quotes
1. Access to care a. Availability and appropriateness of care b. Time to access care	Lack of availability of healthcare providers and distance to services an issue in receiving timely access to care, particularly for emergency department and for referrals to specialists. ‘In my mind, uh, we have a pretty darn good health system, the challenge is getting into the system’. (AHS Patient and Family Advisory Member; Group 1, participant 4) ‘The issue was finding a family doctor, there wasn't one available. It was a long wait, but eventually a friend told me about a doctor and through his referral I had finally found a doctor’. (Latino‐Hispanic Community Member; participant 2) ‘When you go to the family doctor, they put you in the room and then you have to wait for half an hour, and when the doctor comes he only talks to you and sees you for five minutes. He might see your blood pressure and he might not. I am sick and tired, and I wanted to see the doctor and the doctor barely sees me for five minutes?’ (Syrian Community Member; participant 1) ‘We [live] less than 5 miles away from ambulance so we had service at the door within minutes…So, um, in Wetaskiwin we sat up there for a little while then transferred to Edmonton, had an angiogram and angioplasty within 25‐24 hours, so um … so I was extremely fortunate…’ (AHS Patient and Family Advisory Member; Group 2, participant 2)
2. Cost of care a. ‘Free’ healthcare b. Insurance, coverage and benefits	Many participants appreciated that healthcare is ‘free’ in Canada. Coverage is not sufficient to cover dental care, medications and therapies, such as psychologists and physiotherapy. Participants were concerned about the high cost of essential services, such as ambulance services. ‘We have to consider that we are getting this healthcare for free. That's another thing, you know like so many things especially terminal illness many other conditions, so I think we have to keep that in our mind that you are getting good service in terms of monetary/financial conditions. And its same for everyone….’ (South Asian Community Member; participant 5) ‘She was crying in pain, so from there I had to call an ambulance and I got worried because I didn't have any money and I was told that ambulance services cost money and that it was expensive…’ (Latino‐Hispanic Community Member; participant 4) ‘But, after that, when I got a job I was covered. My concern is, if someone is laid off, like now Alberta's situation is very bad, what will happen for those who are laid off? Like if someone is diabetic they have to take a medicine, they cannot afford it to buy by themselves. Like…you cannot afford it to buy it if you are laid off. This is my concern’. (East African Community Member; participant 3)
3. Medical tourism/consultation	Travel or consultation with doctors abroad for care seen as relatively common among ethnocultural communities due to issues related to timely access to care, cost and familiarity with the healthcare system. ‘…the Chinese had a consensus that if we had diagnosed with any kind of diseases and need to wait for a long time, we should also consult (the doctors) back in China. If the doctors suggested doing an operation, you better purchase an air ticket immediately. Life is very precious’. (Chinese Community Member; participant 2) ‘A lot of Canadians that I know go to Mexico for dental treatment because they know its more expensive here’. (Latino‐Hispanic Community Member; participant 5)
4. Communication a. Respectful and compassionate care b. Language barriers c. Sharing sufficient information	There is a need for relationship building between healthcare providers and patients. Issues experienced by participants included language barriers and having sufficient, high‐quality information about treatment and care options. ‘Actually whenever I go to doctor, they call me by name. Once [they] call me by my name I feel close, attached to them. Otherwise, I'm going to feel bad. my relationship with my doctor is really good’. (South Asian Community Member; participant 8) ‘I asked, like, he asked me questions and he doesn't focus with me. There's no that connection… between you. Like he has to listen to you first, but he's on the computer like “uh huh. What happened to you?—Uh huh”. I don't want that. Like, I want, like, personal connection. He has to understand me. What's my pain…and they don't look’ (East African Community Member; participant 7) ‘Patients are not valid in the eyes of many healthcare workers, they have no validity, and therefore they're ignored…so there's this almost talking down to you, and maybe you don't understand me, and I'm thinking, I'm not stupid, do not dumb this down for me’. (AHS Patient and Family Advisory Member; Group 2, participant 6) ‘There is one lady I know. She doesn't speak English; they didn't offer a translator. In the hospital, they ask her “do you want a pain killer?” She said no because she didn't understand. She was in pain all her delivery…’ (Syrian Community Member; participant 6)
5. Patient and caregiver engagement	Patients value that healthcare providers engaged them and their families as part of their care team. They want to be included in making decisions about their healthcare and to discuss their preferences and expectations for their care ‘Ya, so, being a valued member of the care team, and to be treated as a human being, versus just this patient… cause I had a lot of experience with that’. (AHS Patient and Family Advisory Member; Group 1, participant 5) ‘Yeah, I guess what I would value most is the collaborative relationships for making healthcare decisions’. (AHS Patient and Family Advisory Member; Group 2, participant 2) ‘…just like my case that I underwent surgery twice. They won't allow my family member…to stay overnight’. (Filipino Community Member; participant 3)
6. Preferences and expectations for care	Preferences included having discussions about various treatment and care options as well as having an understanding of patients' culture and/or language. There were expectations around follow‐up and continued care, but some experienced a sense of ‘you're on your own’. ‘Religiously we don't have a problem, but culturally no Somali female wants to face a man’. (East African Community Member; participant 4) ‘I think that the physiques between Asian and North American women are different. They do not have concepts like “sitting the month” (postpartum confinement). I think that Asian women are very fragile as they insist on natural delivery if all possible’. (Chinese Community Member; participant 2) ‘Culturally what we consider an emergency for our kids might not be considered an emergency here’. (Latino‐Hispanic Community Member; participant 4)
7. Equality of care	Discrimination experiences in care: unequal treatment due to different coverage (private vs. Alberta Health), immigration status, language barriers and gender identity or sexual orientation. These experiences discouraged participants from seeking care. ‘The system here is really good. Our representatives go to the same hospital as everybody, to the same hospital we go to. It's a really good thing that the minister will lineup the way we do’. (Syrian Community Member; participant 2) ‘She was very rude and it seemed like she didn't want to help me because I was a foreigner and because I didn't speak English’. (Latino‐Hispanic Community Member; participant 1)
8. Integrated models of care	Patients value centralized and coordinated care where services are located in the same area and that there is communication between different healthcare providers who patients may interact with ‘… it is a reactive system rather than a preventative system. I had looked forward to being more preventative, being proactive, knowing what she had gone through so if we gradually start getting care for her and support for her…’ (AHS Patient and Family Advisory Group; Group 2, participant 7) ‘So, that's one thing I really appreciate, like everything is centralized, you know. I go to any doctor, they just have to open my file and probably see the all history, right. I don't have to keep doing the same test again and again for each and everything’. (South Asian Community Member; participant 2)
9. Patient safety	Situations of mismanaged care have led to poor health outcomes including death. M5: ‘They made a mistake and they cut some stuff inside his stomach…’ M: ‘And so how do you know they made a mistake?’ M5: ‘Well, the doctor said…’ M3: ‘The doctor said…’ M5: ‘Yeah, yeah. They admitted that’. (East African Community Members; participants 3 and 5) ‘If you have cancer by the time you get diagnosed your cancer would have spread to the level where you can't be treatable. I had a friend, he is from my background, by the time he was diagnosed it took him almost eight months here, like stomach pain, start with that the doctor gave him somethings, after 3 or weeks he went again the same. By the time diagnosed it was too late for the treatment’. (South Asian Community Member; participant 7)
10. Professional ethics a. Informed consent b. Patient rights	Participants wanted healthcare providers to ensure that they always ‘do the right thing’, trying to do what is best for their patients. Issues were discussed regarding informed consent and patient rights. ‘I had a surgery and the doctor that operated on me I met the day of the surgery. They didn't even give me an appointment to meet him or for me to be more informed of the surgery. I only had further attention about my condition after the surgery with my family doctor. After the surgery I never saw the surgeon again’. (Latino‐Hispanic Community Member; participant 6) ‘Doing the right thing is quality, right thing is a standard. So, if you are diagnosed with particular disease for a patient, then you have to do the right things, what you need to do, so quality, in my opinion he's doing the right things’. (South Asian Community Member; participant 3)

While these themes provide insight into the diversity of experiences and perspectives, four were especially salient across all FGDs: access to care, cost of care, communication and patient and caregiver engagement. These themes were thus prioritized for informing the development of the PC‐QIs^13^ and are presented here.

### 3.2 Access to care

Participant discussion related to access to care was frequent and often spoken of with respect to timeliness of care. We capture this using two subthemes: (1) availability and appropriateness of care and (2) long wait times.

1. *Availability and appropriateness of care*: Participants described challenges with a lack of healthcare providers, especially doctors, relative to the large volume of patients. Some reported challenges in being able to find a family doctor (including those who speak their language and share their culture). Participants described feeling rushed at visits with their family doctor, or when unable to access their doctor, being left with a sense of ‘you're on your own’. Participants also cited lack of availability of family doctors outside of work hours as a barrier to accessing care. At the same time, participants appreciated telephone consults with their healthcare providers and the 811 service (24/7 nurse advice and general health information). Participants also discussed how distance to healthcare services affected their experiences, with some appreciating the convenience of the services close to them, while others, particularly those from rural communities, described distance as a barrier to timely and appropriate care.

2. *Long waiting times*: Waiting times represented a major concern for many participants, particularly waits to see a specialist or schedule a surgery (i.e,. waiting 3 months to over a year), accessing emergency services and waiting at the family doctor/walk‐in clinic or laboratory (i.e., 1–4 h), even though appointments had been made. Some participants reported or joked about measures to avoid long waiting times, including ‘pretending to be dying’, using ambulance services even if not necessary, seeking care in other provinces or countries or even not seeking/avoiding care. Some participants shared positive experiences of receiving timely care and having a good health outcome and/or feeling satisfied with their care visit.

Relative to other communities, members of the Chinese community shared more experiences of travelling to China to receive care due to a lack of coverage to access services in Canada or to see specialists more quickly. Some participants spoke of having greater trust in healthcare professionals in China (perceived competency, quality and familiarity with language and culture). Many potential participants from the Chinese community were not eligible to participate in the study as they never accessed Alberta's publicly funded health services, but had instead only accessed Traditional Chinese Medicine healthcare providers.

#### 3.2.1 Cost of care

Our cost of care theme often overlapped with issues of access to high‐quality public care and included issues of services not covered publicly or by insurance. Participants providing evidence of this theme were mainly from ethnocultural communities rather than from the AHS Patient and Family Advisory Group. We organize this theme into two subthemes: (1) ‘free’ healthcare and (2) insurance, coverage and benefits.

1. *‘Free’ healthcare*: Participants valued Canada's ‘free’ healthcare (publicly funded hospital and physician services, some additional services) and expressed appreciation for the care provided. Many participants shared positive perceptions of and experiences with the healthcare system in Canada; they valued everyone having access to care regardless of socioeconomic status or whether they were born in Canada. On the other hand, participants expressed how ‘free’ healthcare can cause health system inefficiencies; they compared their experiences to other healthcare systems where people can pay for healthcare and receive efficient and high‐quality care.

2. *Insurance, coverage and benefits*: Many participants felt that health coverage should be extended, especially for dental care, vision care and prescriptions (varying coverage), and that those who are not working (and not receiving benefits/additional coverage) cannot afford some health services. Even those with additional coverage/benefits from work feel that the coverage is often insufficient. Participants expressed worry about needing to pay for the ambulance—even hesitating to use them—and were surprised to find high bills (upwards of $800) as a result of using the ambulance. Participants considered ambulance a basic emergency service. Participants in the East African FGD expressed concern over patient rights and had experienced challenges with Workers' Compensation Board coverage that included being denied time off to heal from an injury, despite having paid for coverage.

#### 3.2.2 Communication

Many participants' descriptions contribute to our theme of Communication. Participants shared positive and negative experiences that reflected what type of communication they value when interacting with their healthcare providers. We organize communication into three subthemes: (1) respectful and compassionate care; (2) language barriers; and (3) providing and sharing information.

1. *Respectful and compassionate care*: Participants expressed valuing interactions with their healthcare providers where they feel respected. This includes being addressed by name and having healthcare providers share information. Providers who withhold information leave an impression of believing that the patient will not understand what they are being told. Participants conveyed care that was compassionate, empathetic or kind including speaking to the patient and caregivers, treating you like a ‘human being’ and showing that they care—not limiting the interaction to discussions about the disease/treatment. A compassionate interaction also meant asking about how the patient is doing, listening and responding and trying to understand patients' situations that can include experiences of loss, worry of loss of employment, chronic pain and time spent waiting for treatment or to see a specialist. Many participants saw relationship or rapport building as an important aspect of the care that they receive to feel like they trust their healthcare provider. Additionally, participants also discussed the importance of acknowledging gender identity or sexual orientation. Some participants reported experiences with discrimination on the basis of their language or sexual orientation as discouraging them from seeking care.

2. *Language barriers*: Participants shared experiences of language barriers when trying to communicate with healthcare providers or support others with language barriers. Language barriers resulted in not being able to communicate symptoms or fully understand treatment (implications for informed consent). Further, participants described missing appointments because they had not understood what they were told over the phone. A number of participants appreciated having healthcare providers who could speak their language or having interpretation services available, preferably in‐person services.

3. *Providing and sharing information*: Participants viewed receiving sufficient information about treatment and care options (e.g., medication, procedures, instructions posthospitalization) to be important, especially for surgeries, which often leave people feeling anxious. When receiving information about test results, many—although not all—participants did not appreciate the ‘no news is good news’ approach, where they would not be communicated with unless there was a concern. Some said that this approach left them anxious or stressed as they waited to be contacted.

While some participants expressed valuing confidentiality with their healthcare provider, many spoke about wanting to more easily access their health information. They felt that they should have the right to their own information and having access would help to improve the continuity and integration of their care, such that information would not be lost from one care provider to the next. Participants from the AHS Patient and Family Advisory spoke about a number of system‐level challenges around communicating health information. These included an inability to access their own electronic medical records online through a patient portal. Participants also noted that having access to their own health records would empower patients in their care.

#### 3.2.3 Patient and caregiver engagement

Related to the theme of communication, participants described valuing feeling engaged by their healthcare providers. Participants in the AHS Patient and Family Advisory Group emphasized the importance of including the patient and their caregivers as part of the care team, discussing their preferences and expectations and, more broadly, supporting them to participate in making decisions about their health. Participants valued advocates and support systems for helping make their care experiences more positive. Advocates and support systems helped communicate with/receive information from healthcare providers and to accompany patients and families on their care journey. Participants shared experiences of feeling that the healthcare system did not accommodate their caregivers/support systems, for example, by not providing a place to sleep when staying overnight in the hospital. Also, participants shared experiences of being a caregiver/support and not feeling recognized by the healthcare provider as a person who can contribute to decisions around the patient's care.

### 3.3 Knowledge mobilization: Codesigning improvements for PCC

#### 3.3.1 Report back to the community

More than 60 people participated in the ‘report back to the community event’, of whom 38 were study participants. Both ActionDignity and AHS were instrumental in engaging participants and other members of the community to participate in the event. Other participants in attendance included ActionDignity members and community stakeholders. Using ‘dotmocracy’, each participant was given a maximum of five coloured dots for voting on their priority areas. Six of the 10 action areas were prioritized in the following descending order, according to the number of votes:

1. Timely access to care (30 votes)
2. Culturally accessible language (24 votes)
3. Patient and caregiver engagement (17 votes)
4. Respectful and compassionate care (16 votes)
5. Patient rights (16 votes)
6. Cost of care (16 votes)

Participants agreed that patient and caregiver engagement could be addressed in conjunction with respectful and compassionate care (combined into a single action area). With 16 votes each for respectful and compassionate care, patient rights and cost of care, participants decided to prioritize all six, rather than limit the priorities to only five, as originally planned.

#### 3.3.2 Provincial forum on PCC

A total of 111 participants attended the provincial dissemination event, while the workshop session involved a smaller group of stakeholders (total of 38), comprised of patients, community members, the Ministry of Health and AHS leadership and researchers (in PCC, quality improvement and policy). A summary of the prioritized FGD themes (based on the report back to community event), opportunities to address the gaps identified (through initiatives that Alberta stakeholders could implement), considerations for implementation and assessment of feasibility for implementation is shown in Table 3.

**Table 3:** Prioritized themes, opportunities and considerations for implementation

Theme	Proposed opportunities	Considerations for implementation	Feasibility assessment
1. Patient rights	Information sharing and dissemination Having patient and healthcare provider rights accessible	Show on flatscreens in clinics and hospitals, make print‐outs available Include in Healthcare 101 (iKnow Health) a provincial initiative to support Albertans in navigating the healthcare system May help to promote respectful and compassionate care, engagement	Low difficulty
2. Respectful and compassionate care 3. Patient and caregiver engagement	Codesign innovative models of training and evaluation of healthcare providers	Potential role of Patient and Family Advisory Groups and community members/organizations to evaluate performance on providing respectful and compassionate care and demonstrate the codesign model of care	Medium difficulty
4. Culturally accessible language	Provide options for communication needs—assess whether needs include materials in different languages, in‐person interpretation or language line	Provide complementary services based on individual needs (not one service or another) Collaborate with community organizations to identify people who can be interpreters and/or help to translate information	Medium difficulty
5. Timely access to care	Look to improve integration of care processes to address issues related to timely access to care	Implement measures to use at transitions of care, i.e., measure developed by health quality council	Medium difficulty
6. Cost of care	Educate people on what is considered part of the healthcare system and what is not to address expectations	Does not address the issue of cost as a barrier to access and seeking care Requires assessment of what is considered ‘essential’, e.g., ambulance, pharmacare, dental	Challenging

While most initiatives were deemed ‘low’ or ‘medium’ difficulty for implementation, addressing cost of care was considered ‘challenging’, as it would require greater investments into public healthcare and what is considered ‘essential’ to cover.

## 4 DISCUSSION

### 4.1 Summary of key findings

We undertook this study to meaningfully engage patients and communities in the development of quality indicators to measure PCC and identify initiatives to improve PCC. Through a partnership with ActionDignity and AHS, diverse patients and community members in Alberta were engaged throughout the research process—in the design of the study, the data collection, analysis, writing and knowledge mobilization. In particular, and consistent with a transformative paradigm, we used participatory action research approaches to engage newcomer communities, who tend not to be represented in research and policy due to linguistic, cultural and financial barriers.

From this study, we found that patients and community members had both negative and positive experiences with the healthcare system in Alberta, and this leaves us with a greater understanding of what matters to them in their healthcare. Across all focus groups, issues of accessing care, particularly timely access to specialist care, affordability of services (i.e., services not covered publicly), enhanced communication with healthcare providers and the system and patient and caregiver engagement, were considered to be important aspects of PCC. Some differences between groups were evident, with ethnocultural community participants expressing greater challenges associated with the costs of care, language and cultural barriers, compared to the AHS Patient and Family Advisory Group member participants, who emphasized the importance of patient and caregiver engagement in care planning. This suggests some differences in healthcare needs between those more likely to experience financial, language and cultural barriers compared to patients and caregivers who are highly engaged in the healthcare system. This highlights the importance of actively engaging populations who may be less likely to participate in healthcare research, as healthcare policy may not adequately address their needs. ‘Action areas’ identified and prioritized by key stakeholders in healthcare in Alberta (patient rights, respectful and compassionate care/patient and caregiver engagement, culturally accessible language, timely access to care and cost of care) were areas of PCC where improvements were feasible, with the exception of cost of care. Cost of care was considered especially challenging, given the provincial budgetary constraints and decisions needed around what would be publicly funded.

### 4.2 Research in context

Our findings are important for several reasons. By focusing our efforts to engage traditionally marginalized communities through a PCC lens, our study sheds light on how we can design initiatives and measure the quality of healthcare based on the perspective of those who are not typically represented in research and policy decisions. By asking diverse communities about their experiences, we were able to gain an understanding of their specific needs, values and preferences in care (key themes of PCC) to help us attain our study objectives. In particular, participants from newcomer communities appreciated receiving what they experienced as ‘free healthcare’, indicating a value for public healthcare. However, participants from across all the FGDs noted limitations to care, which did not cover certain services, such as mental health, dental care, some medications and ambulance services, and pointed to variations in coverage depending on individual insurance. This suggests that patients view healthcare as more holistic compared with what is covered publicly. This idea of holistic health is consistent with a person‐centred perspective on health, as articulated by Ekman et al. and Santana et al.^1^, ^34^ While costs of care were considered one of the main priorities for the implementation and evaluation of PCC, AHS and the Alberta Ministry of Health did not consider this feasible to address. The consequences of this include a continued gap in the provision of PCC, where what is important to patients remains unaddressed (i.e., healthcare services remain inaccessible). Addressing this gap requires the political will to tackle inequitable access to care. Indeed, our findings fit with calls to expand the publicly funded basket of services in Canada.^35^

Our findings also highlight a need for addressing gaps in the provision of culturally inclusive healthcare as well as care aimed at ensuring that all feel safe, regardless of race, gender, sexual identity and immigration status. Preferences expressed by participants go beyond the need for interpreter services and professionals who share their language and culture. Participant experiences with discrimination in the healthcare system underline the need for greater awareness of patient rights and highlights continued systemic challenges in ensuring equity in the care provided. Moreover, nuances in experiences among different newcomer groups were evident. For example, immigration status (i.e., on a work permit, student status or temporary foreign worker) influenced negative experiences with healthcare due to greater marginalized social positions, which involved pronounced language barriers, lack of familiarity with the healthcare system, employment in precarious work environments and lack of comprehensive health coverage. Similar findings were reported by Woodgate et al., who studied the healthcare experiences of African immigrant and refugee families in Manitoba, Canada,^9^ as well as by Reitmanova and Gustafson, who explored the maternity healthcare needs and the barriers of immigrant Muslim women in accessing health services in St. John's, Canada.^36^ As such, these studies support our own findings and provide a greater context for interpreting them. Despite this study, little research has been carried out to examine these issues and evaluate interventions aimed at addressing discrimination in care, indicating persisting evidence gaps.^37^ Our research contributes towards exploring these issues and calls attention to the need for more. These nuances in experience underscore the importance of PCC, where a patient's specific care needs, values and preferences are taken into account when providing patient care.^1^ A person‐centred model of care necessitates an affirmation of how a patient's context (including cultural, environmental and social factors) influences the care experience.^34^ It will be important to evaluate whether a PCC approach to care will help challenge the structural injustices that hinder patients' access to safe, respectful and compassionate care. PC‐QIs can play a role in evaluating whether PCC addresses these issues and improve the care that diverse and often marginalized patients receive.

Finally, with respect to our second study objective, to use innovative participatory approaches to engage ethnocultural communities in qualitative patient‐engaged research, we acknowledge the importance of our University–community partnership as a key strength of this study. The role of Community Brokers was instrumental for effectively liaising between communities, academia and key stakeholders in health service delivery in Alberta to codesign the PC‐QIs and PCC action items. While the role of community members in linking communities to health services is well established in the literature (as Community/Cultural Brokers, Lay Health Workers, Community Health Workers, etc.), there is very little documented evidence for their role in health research.^38^ Meyer et al. have done important work with training community leaders as researchers and engaging lay health educators to reach out to isolated women in a Hispanic community.^39^ Using these strategies contributed to increased levels of trust and comfort of participants due to shared language and familiarity and greater empowerment of immigrant communities. Meyer et al. also discussed the highly participatory approach as challenging, as it requires considerable time, particularly as a bilingual study.^39^ These findings accord with experiences by Kowal et al.^38^ as well as our own. While we also experienced the challenges of demands on time and resources (e.g., time needed for training and mentorship throughout the study period and for knowledge mobilization, costs associated with holding meetings and honoraria for the Brokers), working with the Brokers and (Organization name) allowed more meaningful engagement with communities that was culturally sensitive and accessible to diverse communities. Furthermore, consistent with a transformative paradigm, the experience proved to be transformational for the research team, key stakeholders and participants, who expressed an appreciation for the process of engagement and the opportunity to mobilize the research findings into potential future actions and initiatives. Our experience leaves us convinced of incredible opportunities ahead for collaborating with community leaders to engage communities more meaningfully in health research and policy.

### 4.3 Limitations

The limitations of our study include multiple approaches of the FGDs (some participants were known to Brokers, some conducted in the native language, and FGDs had different facilitators), which may have influenced our findings. Potential selection bias may have been introduced through our recruitment strategies using convenience sampling (i.e., some participants were known to Brokers). At the same time, our study objectives ensure a diversity of perspectives and we remained flexible in recruitment methods to promote a participatory approach. We also recognize the limitations of focusing on six ethnocultural communities for our FGDs and potential limitations in representativeness of having only 6–12 participants representing their cultural groups. As such, our study findings may not adequately represent the diversity of perspectives from the wider ethnocultural community and may thus limit the applicability of our findings to other ethnocultural groups that were not represented. It is also important that as researchers whose racial/cultural identities are similar to many of the research participants, we acknowledge potential for our own biases influencing the analyses and interpretation of the findings. Despite these limitations, we are confident in the credibility of our findings, given our members checking and obtaining feedback on the results during two dissemination events. Finally, our study is limited by the lack of meaningful engagement of Indigenous communities. While there was Indigenous representation in the AHS Advisory Group, we recognize that Indigenous perspectives are underrepresented in healthcare research and policy, including our own. As such, the applicability of our findings may be limited for Indigenous communities, who may have different perspectives of PCC and the prioritization of the themes identified.

### 4.4 Future research

The next steps for our research involve implementing this evidence into practice. The FGD themes for PCC that have been identified through this study have served as one of the foundational elements for developing PC‐QIs that are informed by the patient perspective. The final PC‐QIs that were developed have been published elsewhere.^13^ With regard to the implementation of PCC initiatives developed through our stakeholder engagement, many of the recommendations identified for improving PCC will be incorporated into the provincial ‘iKNOW Health’ initiative^40^ (formerly ‘Healthcare 101’), of which ActionDignity is a key stakeholder, to ensure that perspectives of ethnocultural and immigrant communities are considered within AHS programmes. Moreover, engagement with patients and communities has been sustained through the process of developing the PC‐QIs, where Community Brokers, members of the AHS Patient Advisory Group and community‐based organizations have been engaged through a consensus process to develop and refine the PC‐QIs.

The application of the Community Broker approach for health research is still novel and requires further research and evaluation. Future research should also involve more meaningful engagement of Indigenous communities, particularly using culturally appropriate participatory approaches that ensure Indigenous‐led perspectives throughout the research process.

## 5 CONCLUSIONS

Addressing access and cost of care, language barriers and culture are important aspects of PCC, according to ethnocultural communities, while patient and caregiver engagement were most important for PCC for members of the provincial Patient and Family Advisory. These themes provide a basis for developing PC‐QIs that will evaluate and improve the quality of care for diverse patients as well as to implement initiatives to address gaps in PCC.

Incorporating patient, caregiver and impacted community voices requires addressing potential issues related to equity and understanding the barriers to effective and meaningful engagement. Partnering with key stakeholders and implementing effective participatory approaches to engaging diverse communities were instrumental in enabling us to address these issues and work together towards codesigning a more person‐centred model of care for the province.

## CONFLICT OF INTERESTS

The authors declare that there are no conflict of interests.

## ETHICS STATEMENT

Ethics approval was obtained from the Conjoint Health Research Ethics Board at the University of Calgary. Informed consent was obtained from all individual participants in the study.

## AUTHOR CONTRIBUTIONS

Maria J. Santana and Kimberly Manalili conceived the study. All authors were involved in the study design, with Bonnie Lashewicz providing expertise regarding qualitative protocol development and design of the interview guide. Kimberly Manalili, Maria J. Santana, Fartoon M. Siad and Marichu Antonio designed the research training for the Community Brokers. Kimberly Manalili, Fartoon M. Siad and Maria J. Santana were involved in data collection and provided research mentorship and support to the Community Brokers. Kimberly Manalili and Fartoon M. Siad led data analysis, with ongoing support by Maria J. Santana. Kimberly Manalili synthesized the findings and coordinated the knowledge mobilization events. Kimberly Manalili, Fartoon M. Siad, Maria J. Santana and Marichu Antonio facilitated members' checking and presented the study's findings to stakeholders. All authors contributed to the interpretation of the data, manuscript development and critical revision of the manuscript for important intellectual content. All authors read and approved the manuscript and agreed to act as guarantors of the work.

## Supporting information

Supporting information.

## Data Availability

The data sets used and/or analysed during the current study are available from the corresponding author on reasonable request.
