# Supplementary material for: Codesigning person‐centred quality indicators with diverse communities: A qualitative patient engagement study
Source: Health Expect. 2021 Dec 2;25(5):2188–202. doi: 10.1111/hex.13388 (PMC9615079; doi:10.1111/hex.13388)
Supplement: Supplementary file 1 — Supporting information. [file HEX-25--s001.docx]

**Focus Group Discussion Guide**

1. Please tell us about your experiences with the healthcare system

*Probes:* What kinds of services have you accessed? Have you had positive, negative, or mixed experiences with your healthcare?; Ask if anyone needs clarification about what is meant by healthcare system

1. Thank you for sharing your (positive, negative, mixed) experiences. What do you see as factors that have shaped your experiences (positive, negative, mixed)?

*Probes:* what is the healthcare system/healthcare providers doing well, what are challenges or barriers?

1. What kinds of things do you value in your healthcare? What matters to you?

*Probes:* how healthcare service providers interact with you, processes of care, access to healthcare

1. Do you have any questions for us? Or is there anything you would like to add about your experiences with the healthcare system?
